# Serum Uric Acid in Chronic Kidney Disease as a Marker of Risk or a Modifiable Driver of Progression: A Systematic Review

**DOI:** 10.7759/cureus.113826

**Published:** 2026-08-02

**Authors:** Daniel E Cook, Syed Sajjad Haider Gardezi, Esha Sharma, Joshua James, Shivam Singla, Fawad Haroon, Bhavna Singla, Allah Bux Ali

**Affiliations:** 1 Internal Medicine, Avalon University School of Medicine, Willemstad, CUW; 2 Internal Medicine, University of Health Sciences, Lahore, PAK; 3 Internal Medicine, Wroclaw Medical University, Wrocław, POL; 4 Internal Medicine, Rehman Medical College, Peshawar, PAK; 5 Internal Medicine, TidalHealth Peninsula Regional, Salisbury, USA; 6 Internal Medicine, Erie County Medical Center, Buffalo, USA; 7 Internal Medicine, Sir Ganga Ram Hospital, Lahore, PAK

**Keywords:** albuminuria, chronic kidney disease, estimated glomerular filtration rate, gout, hyperuricemia, kidney disease progression, renal outcomes, serum uric acid, urate-lowering therapy, xanthine oxidase inhibitors

## Abstract

Serum uric acid has been investigated as a marker and potential therapeutic target in chronic kidney disease (CKD), but its clinical significance remains uncertain because hyperuricemia may represent both a consequence of impaired renal clearance and a possible contributor to kidney injury. This systematic review evaluated associations between serum uric acid status, urate-lowering interventions, and renal outcomes in adults with CKD or hyperuricemia-associated kidney disease. PubMed/MEDLINE, Scopus, and Web of Science were searched for studies published from January 1, 2019, to April 1, 2026, with supplementary citation searching conducted during revision. Seven studies met the eligibility criteria, including randomized controlled trials, trial sub-analyses, exploratory analyses, and post-hoc analyses. Narrative synthesis was performed because clinical and methodological heterogeneity precluded meta-analysis. Higher baseline or sustained serum uric acid was associated with less favorable renal trajectories in selected populations, particularly when assessed longitudinally or as average exposure during follow-up. However, pharmacologic urate reduction did not consistently improve albuminuria, proteinuria, measured glomerular filtration rate, or estimated glomerular filtration rate outcomes. In Preventing Early Renal Loss in Diabetes (PERL), the between-group difference in measured glomerular filtration rate with allopurinol versus placebo was 0.001 mL/min/1.73 m² (95% confidence interval: -1.9 to 1.9; p=0.99). In the Controlled Trial of Slowing of Kidney Disease Progression from the Inhibition of Xanthine Oxidase (CKD-FIX), the corresponding difference in annual estimated glomerular filtration rate decline was -0.10 mL/min/1.73 m² (95% confidence interval: -1.18 to 0.97; p=0.85). These findings support serum uric acid as a context-dependent renal risk signal but do not justify urate-lowering therapy solely for universal renoprotection. Future studies should prioritize serial urate assessment, phenotype-specific analyses, earlier disease stages, intracellular urate biology, and hard kidney outcomes to determine when uric acid represents a marker, mediator, or genuinely modifiable target in CKD progression.

The objective of this systematic review was to evaluate the relationship between serum uric acid status, urate-lowering interventions, and renal outcomes in adults with CKD or hyperuricemia-associated kidney disease. Specifically, this review aimed to synthesize evidence on whether baseline, longitudinal, or average serum uric acid levels and pharmacologic urate reduction are associated with CKD progression, estimated glomerular filtration rate (eGFR) decline, albuminuria, proteinuria, or kidney failure-related outcomes. By integrating prognostic and interventional evidence, this review seeks to clarify whether serum uric acid should be understood primarily as a renal risk marker, a potential disease mediator, a modifiable therapeutic target, or a context-dependent combination of these roles.

## Introduction and background

Chronic kidney disease (CKD) is a progressive and heterogeneous condition characterized by gradual loss of renal function, increased cardiovascular risk, and substantial long-term morbidity. Although established predictors, such as baseline estimated glomerular filtration rate (eGFR), albuminuria, diabetes, hypertension, and cardiovascular disease, remain central to risk stratification, there is continued interest in identifying additional biomarkers that may refine prognostic assessment [1,2]. Serum uric acid has received particular attention because hyperuricemia is common in CKD and is associated with impaired renal excretion, metabolic dysfunction, vascular injury, oxidative stress, endothelial dysfunction, and inflammation. However, whether uric acid is primarily a consequence of declining kidney function or an active contributor to renal deterioration remains a subject of ongoing debate [3].

The biological plausibility linking uric acid to kidney disease progression is supported by several mechanistic pathways. Elevated uric acid may contribute to renal microvascular injury, activation of the renin-angiotensin system, glomerular hypertension, oxidative stress, and tubulointerstitial inflammation [4]. At the same time, CKD itself promotes hyperuricemia through reduced filtration and altered tubular handling of urate. This bidirectional relationship makes interpretation challenging. A high serum uric acid level may reflect a harmful biological process, reduced renal clearance, metabolic burden, or a combination of these factors [5]. Therefore, its clinical meaning depends heavily on context, including CKD stage, proteinuria, diabetes, gout, medication exposure, and the persistence of hyperuricemia over time [3,5].

Clinical evidence has also remained mixed. Some studies suggest that higher baseline or sustained serum uric acid is associated with faster eGFR decline or less favorable renal trajectories [6,7]. However, observational association does not establish therapeutic causality. Elevated serum uric acid may reflect reduced renal clearance, metabolic comorbidity, medication exposure, or other factors that independently contribute to CKD progression. Consequently, lowering uric acid may not improve renal outcomes when hyperuricemia is primarily a marker or downstream consequence rather than a causal driver. Interventional studies have not consistently demonstrated improvements in albuminuria, proteinuria, or eGFR-based outcomes following urate reduction [4,5]. This discrepancy highlights the distinction between serum uric acid as a prognostic marker and as a modifiable therapeutic target. It also suggests that isolated baseline measurements may be insufficient to characterize cumulative urate exposure, whereas longitudinal or average serum uric acid may provide additional prognostic information in selected populations [6-8].

## Review

Materials and methods

Study Design and Reporting Framework

This systematic review was designed to evaluate the relationship between serum uric acid status, urate-lowering interventions, and renal outcome deterioration in adults with chronic kidney disease (CKD) or hyperuricemia-associated kidney disease. The review was structured according to the Population, Intervention/Exposure, Comparator, and Outcome (PICO) framework and reported in accordance with the Preferred Reporting Items for Systematic Reviews and Meta-Analyses (PRISMA) guidelines [9,10]. The population of interest included adults with CKD, CKD with hyperuricemia, or gout with reported kidney outcomes. Eligible exposures included baseline, longitudinal, average, or achieved serum uric acid levels. Pharmacologic interventions included the xanthine oxidase inhibitors allopurinol, febuxostat, and topiroxostat; the uricosuric agent verinurad administered with allopurinol; and empagliflozin, evaluated in an exploratory analysis for its urate-lowering effect. Comparators included lower versus higher serum uric acid levels, placebo, standard care, allopurinol alone, or an alternative urate-lowering therapy. Outcomes included estimated glomerular filtration rate (eGFR) decline, eGFR slope, albuminuria, proteinuria, CKD progression, and kidney failure-related outcomes. No single drug was prespecified as the principal intervention because the review aimed to assess whether urate reduction, across different pharmacologic mechanisms, was associated with improved renal outcomes.

Search Strategy and Data Sources

A systematic search was conducted in PubMed/MEDLINE, Scopus, and Web of Science for studies published from January 1, 2019, to April 1, 2026. The search strategy combined terms related to serum uric acid, hyperuricemia, urate-lowering therapy, chronic kidney disease, and renal outcome progression using Boolean operators. The complete search terms, Boolean structure, databases searched, date limits, and search adaptations are presented in Table 1.

**Table 1:** Search strategy used across databases. CKD: chronic kidney disease

Database	Date range	Search terms and Boolean operators	Filters/limits	Notes
PubMed/MEDLINE	January 1, 2019, to April 1, 2026	(“serum uric acid” OR “uric acid” OR “serum urate” OR hyperuricemia OR hyperuricaemia OR “urate-lowering therapy” OR allopurinol OR febuxostat OR topiroxostat OR verinurad OR “xanthine oxidase inhibitor”) AND (“chronic kidney disease” OR CKD OR “chronic renal insufficiency” OR gout OR “hyperuricemia-associated kidney disease”) AND (“CKD progression” OR “kidney disease progression” OR “renal progression” OR “eGFR decline” OR “eGFR slope” OR albuminuria OR proteinuria OR “urinary albumin-to-creatinine ratio” OR “urinary protein-to-creatinine ratio” OR “kidney failure” OR “end-stage renal disease” OR “end-stage kidney disease” OR dialysis)	Human adult studies; English-language articles; publication date within specified range	PubMed search structure was used as the core strategy
Scopus	January 1, 2019, to April 1, 2026	TITLE-ABS-KEY terms were adapted from the PubMed strategy using the same three search concepts: serum uric acid/hyperuricemia/urate-lowering therapy AND chronic kidney disease/gout AND renal progression outcomes	Human adult studies; English-language articles; publication date within specified range	Search terms were adapted to the Scopus syntax while preserving the same Boolean logic
Web of Science	January 1, 2019, to April 1, 2026	Topic search terms were adapted from the PubMed strategy using the same three search concepts: serum uric acid/hyperuricemia/urate-lowering therapy AND chronic kidney disease/gout AND renal progression outcomes	Human adult studies; English-language articles; publication date within specified range	Search terms were adapted to the Web of Science syntax while preserving the same Boolean logic

Eligibility Criteria

Studies were selected according to predefined inclusion and exclusion criteria focused on adult chronic kidney disease, hyperuricemia-associated kidney disease, serum uric acid exposure, urate-lowering interventions, and renal outcomes. The full eligibility criteria are presented in Table 2.

**Table 2:** Inclusion and exclusion criteria.

Domain	Inclusion criteria	Exclusion criteria
Population	Adult participants with chronic kidney disease, chronic kidney disease with hyperuricemia, or gout with kidney-related outcomes	Pediatric populations
Population	Adults with pre-dialysis kidney disease progression outcomes	Dialysis-only cohorts without pre-dialysis progression outcomes
Population	Patients with native kidney disease	Kidney transplant recipients
Exposure/intervention	Studies assessing baseline serum uric acid, longitudinal serum uric acid, average serum uric acid, or achieved serum uric acid during follow-up	Studies in which uric acid was mentioned only as background information
Exposure/intervention	Studies evaluating urate-lowering therapy with renal outcomes	Studies describing serum uric acid changes without renal outcome assessment
Study design	Randomized controlled trials, active-controlled trials, placebo-controlled trials, exploratory trial analyses, post-hoc analyses, or trial sub-analyses	Case reports, reviews, editorials, commentaries, animal studies, or laboratory studies
Outcomes	Studies reporting estimated glomerular filtration rate change, estimated glomerular filtration rate decline, estimated glomerular filtration rate slope, albuminuria, proteinuria, chronic kidney disease progression, or kidney failure-related endpoints	Acute dietary challenge studies or studies without relevant renal outcome data

Study Selection

After removal of duplicates, titles and abstracts were screened for relevance. Full texts of potentially eligible studies were then assessed against the predefined criteria. Studies were selected if they directly addressed either the prognostic value of serum uric acid or the renal implications of urate-lowering therapy in chronic kidney disease or hyperuricemia-associated kidney disease. This approach allowed inclusion of both prognostic and interventional evidence while maintaining a clear focus on renal outcomes. Supplementary citation searching conducted during revision identified two additional reports, which were assessed using the same predefined eligibility criteria and incorporated into the review. Eligibility was determined solely by the predefined population, exposure or intervention, study design, and renal outcome criteria; the direction, magnitude, or statistical significance of study findings did not influence inclusion.

Data Extraction and Outcomes

Data extraction focused on study design, source trial, population characteristics, sample size, chronic kidney disease status, serum uric acid exposure or urate-lowering intervention, comparator group, follow-up duration, renal outcomes, effect estimates, 95% confidence intervals, p-values, and main findings. The primary outcomes were chronic kidney disease progression, estimated glomerular filtration rate decline, estimated glomerular filtration rate slope, and kidney failure-related outcomes. Secondary outcomes included albuminuria, proteinuria, urinary albumin-to-creatinine ratio, urinary protein-to-creatinine ratio, and change in serum uric acid.

Risk of Bias Assessment and Data Synthesis

Risk of bias was assessed according to study design. The Cochrane Risk of Bias 2 tool was planned for randomized interventional comparisons [11], QUIPS for prognostic serum uric acid analyses [12], and Risk of Bias In Non-randomized Studies of Interventions (ROBINS-I) for post-hoc non-randomized exposure analyses [13]. A narrative synthesis was planned because substantial heterogeneity was expected in study design, population, exposure definition, intervention type, follow-up duration, and renal outcome reporting. Evidence was organized into the following two domains: serum uric acid as a prognostic marker and serum uric acid as a modifiable therapeutic target. This structure was used to avoid conflating prognostic association with therapeutic causality.

Results

Study Selection Process

**Figure 1:**
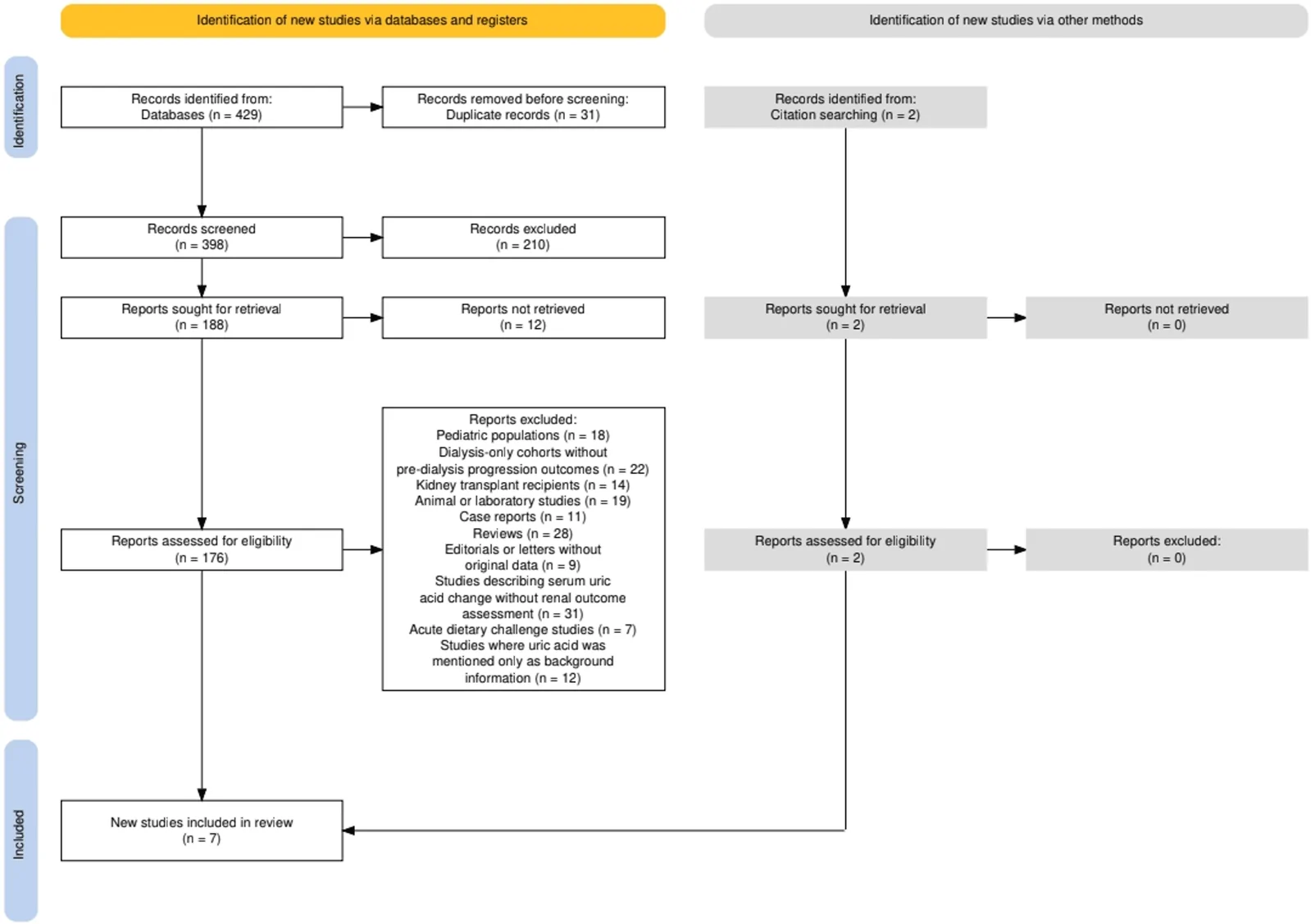
PRISMA flow diagram of study selection. The flow diagram summarizes the identification, screening, eligibility assessment, and final inclusion of studies evaluating serum uric acid status, urate-lowering interventions, and renal outcomes in chronic kidney disease or hyperuricemia-associated kidney disease. PRISMA: Preferred Reporting Items for Systematic Reviews and Meta-Analyses

As shown in Figure 1, the database search identified 429 records, including 146 from PubMed/MEDLINE, 168 from Scopus, and 115 from Web of Science. After removing 31 duplicates, 398 records were screened, of which 210 were excluded. A total of 188 reports were sought for retrieval; 12 could not be retrieved, leaving 176 reports for full-text eligibility assessment. Of these, 171 were excluded for predefined reasons, and five studies met the eligibility criteria. Supplementary citation searching conducted during revision identified two additional reports; both were retrieved, assessed against the same predefined criteria, and included. Therefore, seven studies were included in the final systematic review (Figure 1).

Characteristics of the Selected Studies

As summarized in Table 3, the seven included studies represented a clinically diverse body of evidence evaluating serum uric acid as both a prognostic marker and a modifiable therapeutic target in chronic kidney disease (CKD) or hyperuricemia-associated kidney disease. The included studies comprised randomized trial sub-analyses, exploratory analyses, post-hoc analyses, and randomized controlled trials, with sample sizes ranging from 279 to 6,609 participants and follow-up durations ranging from 24 weeks to three years. Serum uric acid was assessed as a baseline, longitudinal, or average exposure during follow-up or as a therapeutic target through interventions including xanthine oxidase inhibition, verinurad combined with allopurinol, and empagliflozin-associated urate reduction. The populations included patients with CKD, hyperuricemia, gout, type 1 diabetic kidney disease, and stage 3-4 CKD at high risk of progression. Renal outcomes included measured GFR, eGFR decline, eGFR slope, urinary albumin-to-creatinine ratio, urinary protein-to-creatinine ratio, and kidney disease progression outcomes. Collectively, these studies provided complementary evidence on whether serum uric acid functions primarily as a risk marker, a longitudinal renal signal, or a modifiable target for renal outcome improvement.

**Table 3:** Characteristics of included studies evaluating serum uric acid as a prognostic marker and therapeutic target in chronic kidney disease progression. CKD: chronic kidney disease; SUA: serum uric acid; eGFR: estimated glomerular filtration rate; UACR: urinary albumin-to-creatinine ratio; UPCR: urinary protein-to-creatinine ratio; ESKD: end-stage kidney disease; ESRD: end-stage renal disease; SGLT2: sodium-glucose cotransporter 2; EMPA-KIDNEY: Study of the Effect of Empagliflozin on Kidney Disease Progression and Cardiovascular Mortality in Patients With Established Chronic Kidney Disease; ASUCA: Assessment of Clinical Usefulness in CKD Patients With Hyperlipidemia and Hyperuricemia Treated With Atorvastatin; CARES: Cardiovascular Safety of Febuxostat and Allopurinol in Participants With Gout and Cardiovascular Comorbidities Empagliflozin reduced kidney disease progression overall, with no evidence that the treatment effect varied across baseline serum uric acid categories (p for trend = 0.92).

Studies	Design/source trial	Population	Serum uric acid exposure/intervention	Comparator/groups	Follow-up	Renal outcome assessed	Key quantitative finding
Kuwabara et al. (2020) [14]	Sub-analysis of the ASUCA randomized controlled trial	279 Japanese adults with CKD and hyperlipidemia with uric acid data at baseline and 24 months	Baseline serum uric acid and mean serum uric acid during the trial	Serum uric acid groups: <5.0, 5.0-6.0, 6.0-7.0, and ≥7.0 mg/dL	24 months	Change in eGFR after 24 months	Mean eGFR changes across increasing baseline serum uric acid categories were -1.32±10.3, -1.74±8.94, -2.53±7.34, and -3.51±9.10 mL/min/1.73 m², respectively. Multivariable analysis demonstrated an inverse association between baseline serum uric acid and subsequent eGFR change, with a similar trend for mean serum uric acid during follow-up
Mayne et al. (2025) [15]	Exploratory analysis of the EMPA-KIDNEY randomized controlled trial	6,609 adults with CKD	Empagliflozin 10 mg daily; serum uric acid measured at randomization, 2 months, and 18 months	Placebo; subgroup analysis according to baseline serum uric acid	Median: 2 years	Primary composite outcome and kidney disease progression	Empagliflozin reduced serum uric acid by -25.6 µmol/L (95% CI: -30.3 to -21.0). It reduced kidney disease progression overall (HR: 0.71; 95% CI: 0.62-0.81), but this benefit did not differ according to baseline serum uric acid (p for trend=0.92), and on-treatment uric acid did not explain the improvement in chronic eGFR slope
Matsuo et al. (2020) [16]	Multicenter, open-label randomized controlled trial	Adults aged 20-80 years with stage 3-4 CKD, hyperuricemia, eGFR: 15-60 mL/min/1.73 m², and UPCR: 0.15-3.5 g/gCr	Xanthine oxidase inhibitor therapy targeting serum uric acid <6.0 mg/dL	Topiroxostat versus febuxostat	24 weeks	Change in UPCR and eGFR	Serum uric acid decreased from 8.6±1.1 to 6.0±1.1 mg/dL with topiroxostat and from 8.4±1.1 to 5.9±1.3 mg/dL with febuxostat. Median UPCR changes were 0.05 and -0.04 g/gCr, respectively, and eGFR changes were -0.04±4.59 and 0.31±4.70 mL/min/1.73 m²; neither renal outcome changed significantly
Ghang et al. (2025) [17]	Post-hoc analysis of the CARES randomized controlled trial	5,002 adults with gout receiving febuxostat or allopurinol for more than 6 months	Average serum uric acid during urate-lowering therapy	Average serum uric acid <6 versus ≥6 mg/dL	Median: 2.5 years	eGFR slope and risk of eGFR decline	Each 1-mg/dL increase in average serum uric acid was associated with a less favorable eGFR slope (adjusted β = -0.1912). In propensity score-matched analysis, maintaining average serum uric acid <6 mg/dL was associated with lower odds of eGFR decline (adjusted OR: 0.66; 95% CI: 0.57-0.77)
Heerspink et al. (2024) [18]	Phase 2b randomized placebo- and active-controlled SAPPHIRE trial	861 adults with CKD, hyperuricemia, eGFR: ≥25 mL/min/1.73 m², and UACR: 30-5,000 mg/g	Verinurad 3, 7.5, or 12 mg plus allopurinol 300 mg/day	Allopurinol 300 mg/day alone or placebo	34-week primary endpoint; follow-up to 60 weeks	Change in UACR at week 34; UACR, serum urate, and eGFR at week 60	At week 34, geometric mean UACR changes were 16.7% (95% CI: -0.6% to 37.1%), 15.0% (95% CI: -1.85% to 34.6%), and 14.0% (95% CI: -3.4% to 34.4%) across the three combination groups, compared with 9.9% (95% CI: -6.6% to 29.4%) with allopurinol and 37.3% (95% CI: 16.6-61.8%) with placebo. Despite dose-dependent urate reduction, UACR and eGFR did not differ significantly among groups
Doria et al. (2020) [19]	Multicenter, double-blind, placebo-controlled randomized trial	530 adults with type 1 diabetes, serum urate ≥4.5 mg/dL, eGFR 40.0-99.9 mL/min/1.73 m², and evidence of diabetic kidney disease	Allopurinol 200-400 mg/day, adjusted according to kidney function	Placebo	3 years plus a 2-month washout	Baseline-adjusted iohexol-measured GFR after washout; measured GFR slope and urinary albumin excretion	Serum urate decreased from 6.1 to 3.9 mg/dL with allopurinol but remained 6.1 mg/dL with placebo. After washout, the between-group difference in measured GFR was 0.001 mL/min/1.73 m² (95% CI: -1.9 to 1.9; p=0.99). The annual GFR-slope difference was -0.6 mL/min/1.73 m² (95% CI: -1.5 to 0.4), demonstrating no renoprotective benefit
Badve et al. (2020) [20]	Multicenter, double-blind, placebo-controlled randomized trial	369 adults with stage 3-4 CKD and high progression risk; 363 contributed to the primary analysis	Allopurinol 100-300 mg/day	Placebo	104 weeks	Annual change in eGFR and CKD-progression outcomes	Annual eGFR decline was -3.33 mL/min/1.73 m² with allopurinol and -3.23 mL/min/1.73 m² with placebo. The between-group difference was -0.10 mL/min/1.73 m²/year (95% CI: -1.18 to 0.97; p=0.85), despite an adjusted serum-urate reduction of -2.7 mg/dL (95% CI: -3.0 to -2.5)

Risk of Bias Assessment

As shown in Table 4, risk of bias varied across the included studies. RoB 2 was applied to randomized comparisons, QUIPS to prognostic evidence, and ROBINS-I to post-hoc exposure analysis. Overall judgments ranged from low risk to moderate risk, mainly reflecting differences in blinding, exploratory analysis, follow-up duration, and potential residual confounding.

**Table 4:** Risk of bias assessment of studies evaluating serum uric acid and renal outcomes in chronic kidney disease. QUIPS: Quality In Prognosis Studies tool; RoB 2: Cochrane Risk of Bias 2 tool; ROBINS-I: Risk of Bias In Non-randomized Studies of Interventions; CKD: chronic kidney disease; SUA: serum uric acid; eGFR: estimated glomerular filtration rate; UACR: urinary albumin-to-creatinine ratio; UPCR: urinary protein-to-creatinine ratio; EMPA-KIDNEY: Study of the Effect of Empagliflozin on Kidney Disease Progression and Cardiovascular Mortality in Patients With Established Chronic Kidney Disease; ASUCA: Assessment of Clinical Usefulness in CKD Patients With Hyperlipidemia and Hyperuricemia Treated With Atorvastatin; CARES: Cardiovascular Safety of Febuxostat and Allopurinol in Participants With Gout and Cardiovascular Comorbidities; CKD-FIX: Controlled Trial of Slowing of Kidney Disease Progression from the Inhibition of Xanthine Oxidase

Studies	Evidence type in this review	Risk of bias tool applied	Main risk of bias considerations	Overall judgment
Kuwabara et al. (2020) [14]	Prognostic analysis using ASUCA trial data	QUIPS	Serum uric acid categories were not randomized; the post-hoc sub-analysis remained susceptible to residual confounding; only 279 of 344 participants had uric acid measurements at both baseline and 24 months. eGFR was measured objectively, reducing concern regarding outcome assessment	Moderate risk
Mayne et al. (2025) [15]	Exploratory analysis of the randomized EMPA-KIDNEY trial	Cochrane RoB 2	Randomization, placebo control, predefined serum uric acid measurements, and objective renal outcomes supported internal validity. However, the uric acid analyses were exploratory, measurements were unavailable for a substantial proportion of the full trial population, and subgroup analyses may have had limited power to detect interaction effects	Some concerns
Matsuo et al. (2020) [16]	Open-label randomized trial comparing two xanthine oxidase inhibitors	Cochrane RoB 2	Randomization and objective UPCR and eGFR measurements reduced concern regarding outcome ascertainment. The open-label design could have influenced adherence or co-interventions, and the absence of a placebo or non-urate-lowering group limited causal interpretation of renoprotection	Some concerns
Ghang et al. (2025) [17]	Post-hoc non-randomized exposure analysis using CARES trial data	ROBINS-I	Achieved average serum uric acid was not randomized and could reflect adherence, treatment response, gout severity, baseline renal health, and medication exposure. Restriction to participants receiving therapy for more than six months introduced potential selection or survivor bias. Multivariable adjustment and propensity score matching reduced but did not eliminate residual confounding	Moderate risk
Heerspink et al. (2024) [18]	Phase 2b randomized placebo- and active-controlled SAPPHIRE trial	Cochrane RoB 2	Randomization, placebo and active controls, prespecified outcomes, objective UACR and eGFR measurements, and balanced adverse-event reporting supported internal validity. No major concern was identified across the principal RoB 2 domains. The limited duration and reliance on surrogate renal outcomes affected evidence certainty rather than internal risk of bias	Low risk
Doria et al. (2020) [19] - PERL	Multicenter, double-blind, placebo-controlled randomized trial	Cochrane RoB 2	Randomization, allocation concealment, double blinding, placebo control, objective iohexol-measured GFR, prespecified analysis, and balanced follow-up supported internal validity. Treatment discontinuation and missing measurements were addressed through the trial’s longitudinal analytical approach, with no major evidence of differential outcome ascertainment	Low risk
Badve et al. (2020) [20] - CKD-FIX	Multicenter, double-blind, placebo-controlled randomized trial	Cochrane RoB 2	Randomization, blinding, placebo control, and objective eGFR assessment supported internal validity. Recruitment ended below the planned sample size, and treatment discontinuation occurred in approximately 30% of the allopurinol group and 25% of the placebo group. Although the statistical model addressed informative discontinuation, some uncertainty from missing outcome data and treatment adherence remained	Some concerns

Quantitative Synthesis of Renal Outcomes

Serum uric acid as a prognostic marker: In the Assessment of Clinical Usefulness in CKD Patients With Hyperlipidemia and Hyperuricemia Treated With Atorvastatin (ASUCA) sub-analysis, mean changes in estimated glomerular filtration rate (eGFR) over 24 months became progressively more negative across increasing baseline serum uric acid categories. The corresponding changes were -1.32±10.3 mL/min/1.73 m² for serum uric acid <5.0 mg/dL, -1.74±8.94 mL/min/1.73 m² for 5.0-6.0 mg/dL, -2.53±7.34 mL/min/1.73 m² for 6.0-7.0 mg/dL, and -3.51±9.10 mL/min/1.73 m² for ≥7.0 mg/dL. Multivariable analysis demonstrated an inverse association between baseline serum uric acid and subsequent eGFR change, with a similar pattern observed for mean serum uric acid during follow-up [14].

In the post-hoc CARES analysis, 3,264 of 5,002 participants (65.3%) demonstrated a non-negative eGFR slope during urate-lowering therapy over a median follow-up of 2.5 years. Each 1-mg/dL increase in average serum uric acid was associated with a less favorable eGFR slope (adjusted β = -0.1912). In the propensity score-matched analysis, maintaining an average serum uric acid concentration <6 mg/dL was associated with lower odds of eGFR decline compared with concentrations ≥6 mg/dL (adjusted odds ratio: 0.66; 95% confidence interval {CI}: 0.57-0.77) [17].

Renal effects of urate-lowering interventions: In the exploratory Study of the Effect of Empagliflozin on Kidney Disease Progression and Cardiovascular Mortality in Patients With Established Chronic Kidney Disease (EMPA-KIDNEY), empagliflozin reduced serum uric acid by a study-average between-group difference of -25.6 µmol/L (95% CI: -30.3 to -21.0). Empagliflozin reduced the risk of kidney disease progression compared with placebo (hazard ratio: 0.71; 95% CI: 0.62-0.81), but the magnitude of this effect did not differ according to baseline serum uric acid concentration (p for trend=0.92). Exploratory mediation analysis found no evidence that on-treatment serum uric acid explained the improvement in chronic eGFR slope [15].

In the randomized comparison of topiroxostat and febuxostat, serum uric acid decreased from 8.6±1.1 to 6.0±1.1 mg/dL with topiroxostat and from 8.4±1.1 to 5.9±1.3 mg/dL with febuxostat. Despite these reductions, median changes in urinary protein-to-creatinine ratio were 0.05 and -0.04 g/gCr, respectively, and neither change was statistically significant. Changes in eGFR were also non-significant at -0.04±4.59 mL/min/1.73 m² with topiroxostat and 0.31±4.70 mL/min/1.73 m² with febuxostat [16].

In the SAPPHIRE trial, geometric mean changes in urinary albumin-to-creatinine ratio (UACR) at week 34 were 16.7% (95% CI: -0.6% to 37.1%), 15.0% (95% CI: -1.85% to 34.6%), and 14.0% (95% CI: -3.4% to 34.4%) with verinurad 3 mg, 7.5 mg, and 12 mg plus allopurinol, respectively. Corresponding changes were 9.9% (95% CI: -6.6% to 29.4%) with allopurinol alone and 37.3% (95% CI: 16.6-61.8%) with placebo. Although combination therapy produced dose-dependent serum urate reduction, UACR did not differ significantly among treatment groups, and no significant between-group difference in eGFR change was observed through week 60 [18].

In the Preventing Early Renal Loss in Diabetes (PERL) trial, mean serum urate decreased from 6.1 mg/dL to 3.9 mg/dL with allopurinol but remained approximately 6.1 mg/dL with placebo. After three years of treatment and a two-month washout, the baseline-adjusted between-group difference in iohexol-measured GFR was 0.001 mL/min/1.73 m² (95% CI: -1.9 to 1.9; p=0.99). The annual measured GFR decline was -3.0 mL/min/1.73 m² with allopurinol and -2.5 mL/min/1.73 m² with placebo, corresponding to a between-group difference of -0.6 mL/min/1.73 m² per year (95% CI: -1.5 to 0.4) [19].

In the Controlled Trial of Slowing of Kidney Disease Progression from the Inhibition of Xanthine Oxidase (CKD-FIX), annual eGFR decline was -3.33 mL/min/1.73 m² with allopurinol (95% CI: -4.11 to -2.55) and -3.23 mL/min/1.73 m² with placebo (95% CI: -3.98 to -2.47). The between-group difference was -0.10 mL/min/1.73 m² per year (95% CI: -1.18 to 0.97; p=0.85), despite an adjusted serum-urate reduction of -2.7 mg/dL (95% CI: -3.0 to -2.5) with allopurinol relative to placebo [20].

Meta-analysis was not performed because substantial heterogeneity in study populations, serum uric acid exposure definitions, pharmacologic interventions, comparator groups, follow-up durations, and renal endpoints precluded calculation of a clinically valid pooled effect estimate.

Discussion

Synthesis of the Main Findings

The seven included studies indicate that serum uric acid has a complex and context-dependent relationship with renal outcomes in CKD and hyperuricemia-associated kidney disease. Prognostic analyses associated higher baseline or sustained serum uric acid with less favorable renal trajectories, particularly when exposure was assessed longitudinally [14,17]. In contrast, randomized interventional evidence consistently showed that biochemical urate reduction did not necessarily translate into improvement in albuminuria, proteinuria, measured GFR, or eGFR decline [15,16,18-20]. In PERL, allopurinol reduced mean serum urate from 6.1 mg/dL to 3.9 mg/dL, yet the between-group difference in measured GFR after treatment and washout was 0.001 mL/min/1.73 m² (95% CI: -1.9 to 1.9; p=0.99) [19]. Similarly, CKD-FIX demonstrated a between-group difference in annual eGFR decline of -0.10 mL/min/1.73 m² (95% CI: -1.18 to 0.97; p=0.85), despite an adjusted serum-urate reduction of 2.7 mg/dL with allopurinol [20]. These findings do not disprove the biological or prognostic relevance of uric acid, but they demonstrate that an association between hyperuricemia and CKD progression does not establish therapeutic causality. Differences among studies may reflect variation in CKD phenotype, gout status, baseline uric acid, treatment mechanism, timing of intervention, follow-up duration, and outcome selection. Accordingly, serum uric acid may function as a prognostic biomarker, a possible mediator in selected phenotypes, or a therapeutic target when an established clinical indication exists; however, its universal pharmacologic reduction solely to prevent CKD progression is not supported by the evidence synthesized in this review.

Uric Acid as a Prognostic Marker

The prognostic role of serum uric acid is supported most directly by the study by Kuwabara et al. [14]. Across baseline serum uric acid categories of <5.0, 5.0-6.0, 6.0-7.0, and ≥7.0 mg/dL, the corresponding mean changes in eGFR over 24 months were -1.32±10.3, -1.74±8.94, -2.53±7.34, and -3.51±9.10 mL/min/1.73 m², respectively. Multivariable analysis demonstrated an inverse association between baseline serum uric acid and subsequent eGFR change, suggesting that higher uric acid may identify a renal-risk profile associated with progressive functional deterioration. However, the exploratory EMPA-KIDNEY analysis provides an important qualification. Although empagliflozin reduced serum uric acid by -25.6 µmol/L (95% CI: -30.3 to -21.0) and reduced kidney disease progression overall (hazard ratio: 0.71; 95% CI: 0.62-0.81), its renal benefit did not differ according to baseline serum uric acid concentration (p for trend=0.92) [15]. Thus, baseline serum uric acid did not identify patients who derived greater kidney protection from empagliflozin. Taken together, these findings indicate that a single uric acid measurement may provide prognostic information in selected populations but may not consistently stratify renal risk or treatment response across heterogeneous CKD phenotypes. Its interpretation must account for kidney function, tubular handling, medications, volume status, diabetes, and associated metabolic disease.

Longitudinal Uric Acid Exposure as a Stronger Concept

The findings of Kuwabara et al. and Ghang et al. suggest that longitudinal or average serum uric acid exposure may be more prognostically informative than an isolated baseline measurement [14,17]. In the ASUCA sub-analysis, mean serum uric acid during follow-up showed a pattern similar to baseline serum uric acid, with higher values associated with greater eGFR decline [14]. In the post-hoc CARES analysis, each 1-mg/dL increase in average serum uric acid during urate-lowering therapy was associated with a less favorable eGFR slope (adjusted β = -0.1912). Maintaining an average serum uric acid concentration <6 mg/dL was associated with lower odds of eGFR decline than concentrations ≥6 mg/dL (adjusted odds ratio: 0.66; 95% CI: 0.57-0.77) [17]. However, achieved serum uric acid was not randomized and could reflect adherence, treatment responsiveness, gout severity, baseline renal health, or changes in kidney clearance. These findings therefore support longitudinal serum uric acid as a potential risk signal but do not establish that maintaining a specific threshold directly prevents renal decline. Serum uric acid is dynamically influenced by eGFR, tubular secretion and reabsorption, diuretic and SGLT2 inhibitor exposure, dietary factors, insulin resistance, inflammation, and adherence to urate-lowering therapy [4,5,15,17,21,22]. Its clinical value may consequently lie less in an isolated threshold and more in interpreting its trajectory alongside eGFR, albuminuria, comorbidities, and treatment exposure.

Uric Acid as a Therapeutic Target

Interventional evidence does not support serum uric acid as a universal therapeutic target for renoprotection. The SAPPHIRE trial demonstrated that verinurad plus allopurinol produced dose-dependent reductions in serum urate but did not significantly reduce UACR or slow eGFR decline compared with allopurinol alone or placebo [18]. Similarly, topiroxostat and febuxostat substantially lowered serum uric acid without significantly changing UPCR or eGFR over 24 weeks [16]. In PERL, allopurinol reduced serum urate from 6.1 to 3.9 mg/dL, yet the between-group difference in measured GFR was 0.001 mL/min/1.73 m² (95% CI: -1.9 to 1.9; p=0.99) [19]. CKD-FIX likewise found no benefit on annual eGFR decline despite substantial urate reduction (between-group difference: -0.10 mL/min/1.73 m²/year; 95% CI: -1.18 to 0.97; p=0.85) [20].

EMPA-KIDNEY further separated urate reduction from renal benefit as follows: empagliflozin reduced serum uric acid by -25.6 µmol/L (95% CI: -30.3 to -21.0) and reduced kidney disease progression (hazard ratio: 0.71; 95% CI: 0.62-0.81), but the benefit did not vary by baseline uric acid and was not mediated by on-treatment uric acid [15]. In contrast, maintaining average serum uric acid <6 mg/dL in CARES was associated with lower odds of eGFR decline in patients with gout (adjusted odds ratio: 0.66; 95% CI: 0.57-0.77), although achieved urate was not randomized [17]. Collectively, these findings show that biochemical urate reduction does not necessarily produce kidney protection and that any benefit may depend on phenotype, timing, mechanism, and disease stage.

Marker Versus Mediator Versus Modifiable Target Framework

Serum uric acid may function differently according to clinical context. As a risk marker, higher baseline or sustained levels may identify greater renal vulnerability, as suggested by ASUCA and CARES [14,17]. As a possible mediator, uric acid has biological plausibility through oxidative stress, endothelial dysfunction, inflammation, renin-angiotensin activation, and tubulointerstitial injury [4,5]. However, the neutral findings of Matsuo et al., SAPPHIRE, PERL, and CKD-FIX show that lowering serum urate does not consistently improve renal outcomes [16,18-20]. Urate lowering therefore remains appropriate for established indications such as gout, but current evidence does not support its universal use solely to prevent CKD progression.

Phenotype-Specific Interpretation

The relevance of serum uric acid may differ across CKD phenotypes. In gout, sustained urate control is central to treatment and was associated with a more favorable eGFR trajectory in CARES, although residual confounding limits causal interpretation [17]. In proteinuric CKD, neither the SAPPHIRE trial nor the study by Matsuo et al. demonstrated a significant improvement in UACR or UPCR, respectively, despite effective serum urate reduction [16,18]. PERL found no measured-GFR benefit in type 1 diabetic kidney disease [19], while CKD-FIX found no improvement in eGFR slope in stage 3-4 CKD at high risk of progression [20]. EMPA-KIDNEY also showed that SGLT2 inhibitor-associated urate reduction did not explain kidney protection [15,22]. Serum uric acid should therefore be interpreted alongside gout status, CKD cause and stage, proteinuria, diabetes, medication exposure, and longitudinal renal trajectory rather than through a universal threshold.

Why Interventional Trials May Be Negative Despite Biological Plausibility

Several mechanisms may explain why urate-lowering trials have produced neutral renal outcomes. First, elevated serum uric acid may reflect reverse causality as follows: declining filtration and impaired tubular urate handling increase circulating urate, making hyperuricemia a consequence or marker of CKD rather than its primary driver [4,5]. Mendelian-randomization evidence also does not support a causal effect of serum urate on eGFR or CKD risk [23]. Second, treatment may begin after irreversible nephron loss, fibrosis, and vascular remodeling have developed, particularly in advanced CKD. However, timing cannot fully explain the neutral evidence because PERL also found no benefit in early-to-moderate diabetic kidney disease [19], while CKD-FIX was neutral in stage 3-4 CKD [20].

A further possibility is a disconnect between circulating and intracellular urate biology. Serum uric acid does not directly measure urate uptake into renal cells, xanthine oxidase activity, oxidative stress, or established tissue injury; therefore, lowering serum concentrations may not reverse intracellular signaling or structural damage already present [4,5]. Endpoint selection may also contribute, as albuminuria may not capture predominantly tubular, vascular, or interstitial injury. Nevertheless, neutral measured-GFR and eGFR findings in PERL and CKD-FIX indicate that endpoint choice alone is insufficient to explain the absence of benefit [19,20]. Finally, xanthine oxidase inhibition, URAT1 inhibition, and SGLT2 inhibitor-associated uricosuria act through different pathways and should not be assumed to produce equivalent renal effects [15,18,22]. These explanations remain hypotheses and do not establish renoprotection where randomized trials have reported neutral outcomes.

Clinical Implications

Serum uric acid may contribute to renal-risk assessment when elevations are persistent or accompanied by gout, metabolic disease, proteinuria, or progressive eGFR decline [14,17,21]. Repeated or average measurements may be more informative than a single value but should be interpreted alongside eGFR, albuminuria, blood pressure, diabetes, medications, and cardiovascular risk [14,17]. Current evidence does not support initiating urate-lowering therapy solely for renoprotection in unselected CKD patients, particularly given the neutral findings of PERL and CKD-FIX [19,20]. Treatment remains appropriate for established indications such as gout and symptomatic hyperuricemia [4,5]. Although sustained urate control was associated with a more favorable eGFR trajectory in CARES, this non-randomized finding should not be interpreted as proof of kidney protection [17].

Strengths, Limitations, and Future Directions

This review has several strengths, including its integration of both prognostic and interventional evidence, its distinction between serum uric acid as a risk marker and uric acid as a modifiable therapeutic target, and its emphasis on longitudinal urate exposure rather than isolated baseline measurement alone. By including studies such as Kuwabara et al. [14], Mayne et al. [15], Matsuo et al. [16], Ghang et al. [17], and Heerspink et al., this review captures the central clinical tension in this field as follows: serum uric acid may identify patients at higher renal risk, yet pharmacologic urate reduction has not consistently produced measurable renal benefit across CKD populations [18]. However, several limitations should be acknowledged. The included studies differ substantially in design, population, follow-up duration, uric acid assessment, intervention type, and renal endpoints, which limits direct comparability. Some evidence comes from post-hoc or exploratory analyses, and residual confounding remains possible where serum uric acid achievement or longitudinal exposure was not randomized. In addition, outcomes varied from surrogate measures such as UACR, UPCR, and short-term eGFR change to longer-term eGFR slope, while hard kidney endpoints were not consistently reported [19,20]. Future research should prioritize time-updated uric acid models, serial urate trajectories, phenotype-specific analyses, and longer randomized trials powered for sustained eGFR decline, kidney failure, dialysis, and transplantation. Studies should also clarify whether uric acid reduction itself mediates renal benefit or whether it is simply a parallel metabolic signal accompanying broader cardiorenal protection.

## Conclusions

The available evidence supports interpreting serum uric acid as a context-dependent renal risk signal rather than a uniformly causal or modifiable driver of CKD progression. Higher baseline or sustained levels may identify less favorable renal trajectories, particularly when assessed longitudinally or in patients with gout. However, the neutral findings of PERL, CKD-FIX, and other randomized trials demonstrate that substantial pharmacologic urate reduction does not consistently improve albuminuria, proteinuria, measured GFR, or eGFR decline. Urate-lowering therapy should therefore not be initiated solely for renoprotection in unselected CKD patients without another established indication. Clinical interpretation should integrate serial uric acid measurements with eGFR, albuminuria, CKD phenotype, diabetes, blood pressure, medications, and cardiovascular risk. Future research should determine whether earlier intervention, selected phenotypes, or measures of intracellular urate activity can identify patients in whom uric acid represents a genuinely modifiable renal target rather than a consequence of impaired kidney function.
